# Discovery of Cisplatin Binding to Thymine and Cytosine on a Single-Stranded Oligodeoxynucleotide by High Resolution FT-ICR Mass Spectrometry

**DOI:** 10.3390/molecules24101852

**Published:** 2019-05-14

**Authors:** Wenjuan Zeng, Yanyan Zhang, Wei Zheng, Qun Luo, Juanjuan Han, Jian’an Liu, Yao Zhao, Feifei Jia, Kui Wu, Fuyi Wang

**Affiliations:** 1 Beijing National Laboratory for Molecular Sciences, National Centre for Mass Spectrometry in Beijing, CAS Key Laboratory of Analytical Chemistry for Living Biosystems, Institute of Chemistry, Chinese Academy of Sciences, Beijing 100190, China; zengwj2014@iccas.ac.cn (W.Z.); zhangyy0816@iccas.ac.cn (Y.Z.); zhengwei0246@iccas.ac.cn (W.Z.); qunluo@iccas.ac.cn (Q.L.); hjuan@iccas.ac.cn (J.H.); lja@iccas.ac.cn (J.L.); yaozhao@iccas.ac.cn (Y.Z.); poppyjff@iccas.ac.cn (F.J.); 2 University of Chinese Academy of Sciences, Beijing 100049, China; 3 Key Laboratory of Hubei Province for Coal Conversion and New Carbon Materials, School of Chemistry and Chemical Engineering, Wuhan University of Science and Technology, Wuhan 430081, China; 4 Basic Medical College, Shandong University of Chinese Traditional Medicine, Jinan 250355, China

**Keywords:** cisplatin, oligodeoxynucleotide, binding sites, thymine, cytosine, FT-ICR MS

## Abstract

The clinically widely-used anticancer drug, cisplatin, binds strongly to DNA as a DNA-damaging agent. Herein, we investigated the interaction of cisplatin with a 15-mer single-stranded C,T-rich oligodeoxynucleotide, 5′-CCTT_4_CTT_7_G_8_C_9_T_10_TCTCC-3′ (ODN15), using ultra-high resolution Fourier transform ion cyclotron resonance mass spectrometry (FT-ICR MS) in conjunction with tandem mass spectrometry (top-down MS). Top-down MS analysis with collision-induced dissociation (CID) fragmentation of the mono-platinated and di-platinated ODN15 provided abundant and informative Pt-containing or Pt-free a/[a − B], w and internal fragments, allowing the unambiguous identification of T_4_, T_7_, C_9_, and T_10_ as the platination sites on the cisplatin-ODN15 adducts. These results revealed that, in addition to the well-established guanine site, the unexpected thermodynamic binding of cisplatin to cytosine and thymine bases was also evident at the oligonucleotide level. Furthermore, the binding models of cisplatin with cytosine and thymine bases were built as the Pt coordinated to cytosine-N(3) and thymine-N(3) with displacement of the proton or tautomerization of thymine. These findings contribute to a better understanding of the mechanism of action of cisplatin and its preference for gene loci when the drug binds to cellular DNA, and also demonstrate the great potential and superiority of FT-ICR MS in studying the interactions of metallodrugs with large biomolecules.

## 1. Introduction

Cisplatin, a representative of metal-based anticancer agents, is one of the most widely used chemotherapy drugs for clinical treatment against various cancers [1]. DNA is generally accepted to be the primary target for cisplatin, which binds to DNA bases and forms platinum-DNA adducts [2,3]. The binding of cisplatin to DNA often causes changes in the conformation of DNA strands, which subsequently block DNA replication and transcription, initiating various cellular signaling pathways and ultimately triggering apoptosis [1,4,5]. A large number of studies have demonstrated that the binding sites of cisplatin to DNA and the generated structural alteration of DNA duplex play a crucial role in mediating cisplatin cytotoxicity [1,3,6]. Therefore, the accurate localization of DNA binding sites of cisplatin would contribute to better elucidating the mechanism of action of this drug and its preference for gene loci when it binds to cellular DNA.

Cisplatin has been demonstrated to bind to the purine-N(7) of guanine and adenine residues on DNA, mainly forming 1,2-intrastrand crosslinks between adjacent two guanines (1,2-d(GpG), ~65%) or neighboring adenine and guanine (1,2-d(ApG), ~25%), as well as interstrand crosslinks and mono-functional adduct [7,8,9,10]. The order of reactivity of the four nucleotides with cisplatin was established as GMP > AMP >> CMP > UMP/TMP by Raman difference spectrophotometry [11]. Although it seemed that pyrimidine nucleobases have low reactivity to cisplatin, numerous studies have demonstrated the existence of “platinum pyrimidine blues” obtained from reactions of cisplatin and pyrimidine nucleobases such as uracil, 1-methyluracil, thymine, 1-methylthymine, uridine, and thymidine [12,13,14,15,16]. Moreover, Tobias et al., using Raman plus ^1^H-NMR and ^13^C-NMR spectroscopy, observed the coordination of *cis*-[(NH_3_)_2_Pt(H_2_O)_2_]^2+^ to cytidine-N(3) and the deprotonated uridine-N(3) [14]. Lippert et al. found that *cis*-[(NH_3_)_2_Pt(H_2_O)_2_]^2+^ bound to 1-methylthymine at N(3) position yields the bis(1-methylthyminato-N3)*cis*-diammineplatinum(ΙΙ), possibly mediating the tautomerization of thymine [17,18], and the binding could also occur at both N(3) and O(4) positions to form the bis(1-methylthyminato)-bis-(*cis*-diammineplatinum(ΙΙ)) dimers [19]. These observations implied that pyrimidine nucleobases may be also involved in the coordination of cisplatin to DNA. However, previous studies on the reaction of cisplatin and pyrimidine bases have generally used nucleotide models, which are different from the native DNA with secondary and tertiary structures [11,14,17,18,19]. As there are many C-rich or T-rich sequences in genome DNA, it is necessary to study the interaction of cisplatin with pyrimidine bases at the oligonucleotide level.

Many analytical techniques have been employed to characterize the metal complex-DNA interactions, including NMR spectroscopy, X-ray diffraction analysis, and mass spectrometry (MS) [20,21,22,23]. NMR spectroscopy and X-ray diffraction analysis can provide substantial structural information on nucleotide- and other DNA model-cisplatin adducts [20,21,24,25,26,27]. However, the separation and purification of the reaction products as well as the need for a large quantity of the products poses difficulties for the use of these two techniques. With the virtues of high sensitivity, reduced sample consumption, chemical specificity, and ability to handle complex samples coupling with separation techniques, mass spectrometry has become a powerful tool for investigating diverse interactions, especially for the identification of the binding sites of metal complexes on DNA with the development of soft ionization technology and tandem mass spectrometry (MS/MS) [28,29,30,31,32]. Among the various existing mass spectrometry, Fourier transform ion cyclotron resonance mass spectrometry (FT-ICR MS) is unparalleled in terms of resolution (over 1,000,000) and mass accuracy (ppm or sub ppm), providing the highest confidence in spectra assignment and making it ideally suitable for studying the interactions of metallodrugs with DNA [33,34]. Generally, there are two types of mass spectrometric methods for the identification of binding sites: MS analysis of the products arising from nuclease digestion of the metal complex-DNA adducts (termed bottom-up MS) and MS/MS analysis of the intact metallated DNA adducts, which are directly introduced into mass spectrometer and fragmented by various excitation techniques (termed top-down MS) [31,32]. The latter is simpler and more direct without the addition of other agents or adjusting pH.

Single-stranded oligodeoxynucleotides (ODNs) with -GC- in the center, like 5′-CCTT_4_CTT_7_G_8_C_9_T_10_TCTCC-3′ (ODN15), are usually used to form inter-strand crosslinks of cisplatin with their complementary strands. The G_8_ in ODN15 is expected to be the major binding site for cisplatin. However, during the preparation of such ODN adducts, we found more than one cisplatin moiety binding to ODN15, which suggested that T or C might also be platinated besides G. Therefore, in the present work we performed a full characterization of the interaction between cisplatin and the single-stranded ODN15 using high resolution FT-ICR MS, and located the platination sites by top-down MS analysis. In addition to the well-established guanine site, the binding of cisplatin to thymine and cytosine on ODN15 was unambiguously identified. The binding models of cisplatin with thymine and cytosine bases were built, and molecular models of the cisplatinated ODN15 at different sites were also compared.

## 2. Results and Discussion

### 2.1. Interactions of Cisplatin and ODN15

**Figure 1:**
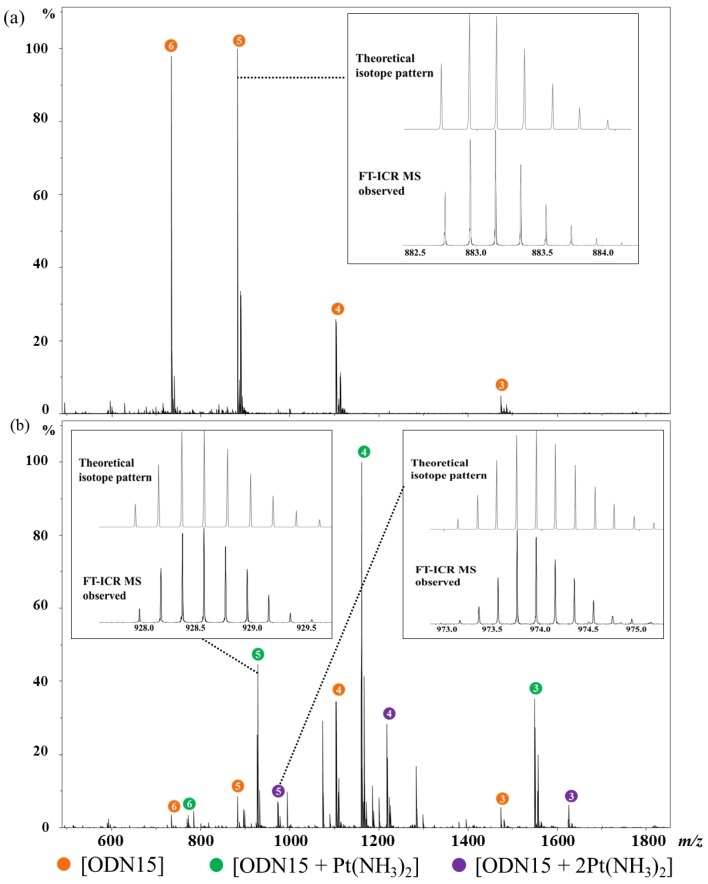
Mass spectra of 5′-CCTT_4_CTT_7_G_8_C_9_T_10_TCTCC-3′ (ODN15) (**a**) and the reaction mixture of ODN15 and cisplatin incubated at 310 K for 72 h (**b**). Mass peaks of unreacted ODN15, mono-platinated ODN15, and di-platinated ODN15 were labeled by orange, green, and purple circles, respectively, with the corresponding negative charge number inserted in each circle. Representative theoretical (**top**) and Fourier transform ion cyclotron resonance mass spectrometry (FT-ICR MS) observed (**bottom**) isotope pattern of [ODN15 − 5H]^5−^ (inset in (**a**)), [ODN15 + Pt(NH_3_)_2_ − 7H]^5−^ (left inset in (**b**)), and [ODN15 + 2Pt(NH_3_)_2_ − 9H]^5−^ (right inset in **b**) are also shown.

Electrospray ionization (ESI) generates ions usually via the addition or removal of H during the desolvation of the charged sample droplets produced from the electrospray needle. Due to the abundant phosphate groups on the backbone, DNA and RNA can produce many multi-charged [M − nH]^n−^ anions in electrospray ionization, even without the aid of basic additives. Accordingly, we applied negative-ion mass spectrometry to investigate the interactions between cisplatin and ODN15. Mass spectrum of free ODN15 was first acquired for comparison (Figure 1a and Table S1 in the Supporting Information). Free ODN15 produced a series of multi-charged ions (3−, 4−, 5−, and 6−) with [ODN15 – 5H]^5−^ being the most abundant one (Figure 1a), and the observed isotope pattern well matched the simulated one (inset in Figure 1a). After reacting with equal molar of cisplatin, mono-platinated ODN15 and di-platinated ODN15 were detected, with the former one having 4− charged adducts [ODN15 + Pt(NH_3_)_2_ − 6H]^4−^ as the main platinated products (Figure 1b and Table S2). Platinum adducts were easily identifiable due to the characteristic isotope pattern of platinum element (inset in Figure 1b). It was apparent that except for the substitution of one chloride ligand by a coordination site on DNA, another chloride ligand of cisplatin was supposed to be lost during electrospray ionization process, which was confirmed by the detected platinum moiety in the platinated adducts. Ammonia ligands were also reported to be lost during the primary MS process when cisplatin interacted with ODNs or proteins [35,36], but this was not been observed in current research. The typical binding sites of cisplatin on DNA are guanine (G) and adenine (A), with G as the major site [37]. However, the ODN15 used in this study contains only one G and no A, so the identification of di-platinated ODN15 adducts suggested a new binding site of Pt, cytosine or thymine.

### 2.2. Identification of Binding Sites of Cisplatin on ODN15 by Top-Down MS

**Figure 2:**
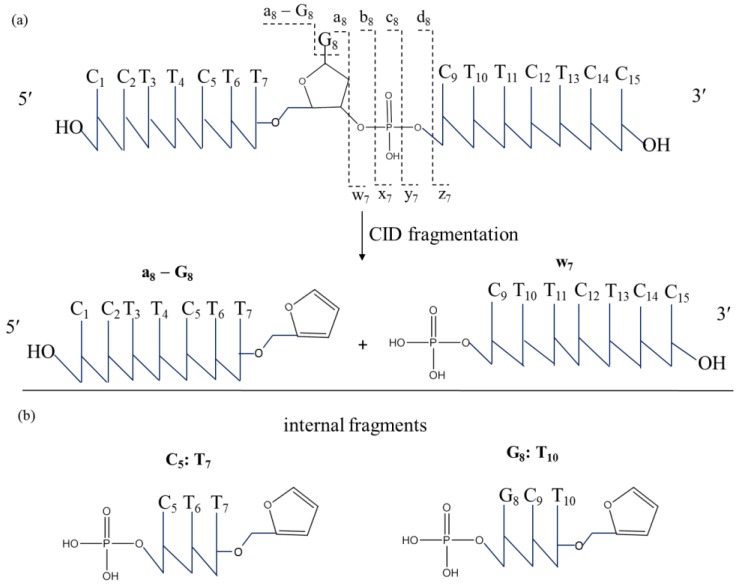
Schematic diagram of tandem mass spectrometry (MS/MS) fragmentation of single-stranded ODN15. Single collision-induced dissociation (CID) fragmentation results in a/[a − B] and w fragments having an intact 5′ terminus and 3′ terminus, respectively (**a**). Internal fragments usually result from double fragmentation at two separate a/w sites (**b**).

To identify the binding sites of cisplatin on ODN15, MS/MS analysis by CID was employed to fragment mono-platinated adduct [ODN15 + Pt(NH_3_)_2_] and di-platinated adduct [ODN15 + 2Pt(NH_3_)_2_] with −5 charges. The commonly applied nomenclature devised by McLuckey et al. for assignment of oligonucleotide MS/MS fragments is shown in Figure 2 [38]. During the CID process, ODNs typically undergo the loss of a nucleobase (A, T, C, or G) and a second elimination reaction, leading to the formation of a furan ring followed by cleavage of the 3′-C-O phosphodiester bond, which yields the complementary [a − B] and w ions with an intact 5′ terminus or 3′ terminus, respectively (Figure 2a) [39,40]. Internal fragments carrying a 5′-phosphate group and a 3′-furan system result from double fragmentation at the separate a/w sites (Figure 2b). Cleavages at other sites along the phosphodiester bond backbone of a DNA strand were significantly infrequent, which simplified the spectra interpretation. MS/MS fragmentation of free ODN15 was firstly acquired to confirm the above mechanism (Figure S1a and Table S3 in the Supporting Information). The fragmentation maps (Figure S1b) and fragments sequence of ODN15 (Figure S1c) indicated excellent 100% cleavage and full coverage for ODN15 sequence during one experimental run. Moreover, we found that w fragment ions were stepped and sequential from w_1_ to w_14_ (Figure S1b,c) whereas a/[a − B] fragments involving neutral base loss were intermittent with no detection of [a_10_ − T_10_], [a_11_ − T_11_] and [a_13_ − T_13_]. This is attributed to the different abundance of each neutral base loss, which is dependent on the relative proton affinity (i.e., C ≈ G > A >> T) and on the position within the oligonucleotides (5′ > 3′ > internal region) [31,41], being more consistent with our observation of the easier loss of cytosine bases than that of thymine bases, especially at the internal region and 3′-terminus of ODN15.

**Figure 3:**
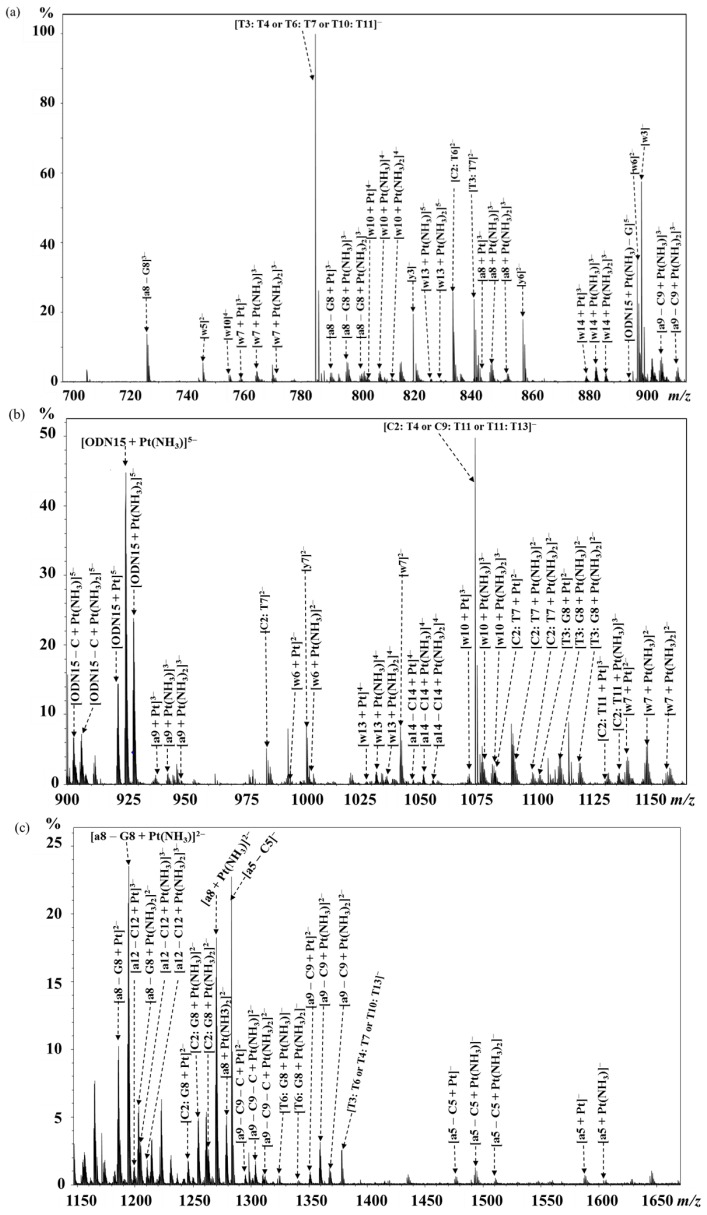
FT-ICR CID MS/MS spectra of [ODN15 + Pt(NH_3_)_2_ – 7H]^5−^ in the mass ranges *m/z* 700–920 (**a**), 900–1165 (**b**), and 1150–1670 (**c**). Main fragments containing platinum or platinum-free are assigned and labeled in the spectra.

**Figure 4:**
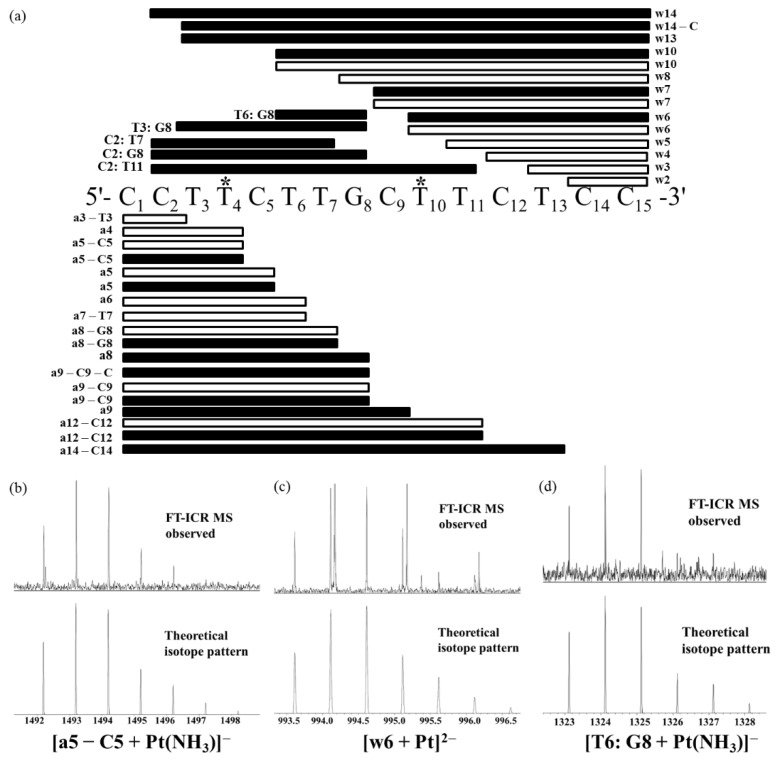
(**a**) Diagrammatic illustration of the localization of the binding sites for cisplatin on ODN15 based on the CID fragments of [ODN15 + Pt(NH_3_)_2_ − 7H]^5−^ observed by MS/MS analysis. Blank box: platinum-free fragments; black-filled box: mono-platinated fragments. * indicates the platination sites deduced by the CID MS/MS data. (**b**–**d**) FT-ICR MS observed (top) and theoretical (bottom) isotope pattern of [a5 − C5 + Pt(NH_3_)]^−^ (**b**), [w6 + Pt]^2−^, (**c**) and [T6: G8 + Pt(NH_3_)]^−^ (**d**).

Next, the mono-platinated ODN15, [ODN15 + Pt(NH_3_)_2_ − 7H]^5−^, was introduced for CID fragmentation (Figure 3 and Table S4). The identified major fragments were w, a/[a − B], and internal fragments similar to those detected for free ODN15. The loss of one or both ammonia ligands of cisplatin results in a characteristic three consecutive neighboring peak clusters of platinated fragments with a mass difference of 17.0265 Da. This facilitated the identification of the platinated fragments, particularly for the low-abundance ones with incomplete isotopic patterns. The platinated fragments with only one ammonium loss were the most abundant peaks among the clusters. From 5′-terminal, no platinum was found to bind at the three bases 5′-C_1_C_2_T_3_, while both Pt-free [a_5_ − C_5_] (observed (obs.) *m/z* 1283.21455 and calculated (calc.) *m/z* 1283.21432 for [a_5_ − C_5_]^−^; obs. *m/z* 641.10372 and calc. *m/z* 641.10352 for [a_5_ − C_5_]^2−^, Figure 3 and Figure 4a) and mono-platinated [a_5_ − C_5_] (obs. *m/z* 1493.19076 and calc. *m/z* 1493.19027 for [a_5_ − C_5_ + Pt(NH_3_)]^−^, Figure 3 and Figure 4a,b) were observed. These indicate that cisplatin bound to T_4_, but not at C_1_, C_2_, or T_3_. However, the detection of larger mono-platinated a_5_, [a_8_ – G_8_], a_8_, [a_9_ – C_9_], a_9_, [a_12_ – C_12_], and [a_14_ – C_14_] fragments (Figure 3 and Figure 4a) suggests that we cannot exclude the binding of cisplatin at the region from C_5_ to C_15_. From the 3′-terminal, no platinated w fragments up to w_5_ were observed, while both Pt-free w_6_ (obs. *m/z* 598.08880 and calc. *m/z* 598.08865 for [w_6_]^3−^, Figure 3 and Figure 4a) and mono-platinated w_6_ (obs. *m/z* 994.61317 and calc. *m/z* 994.61146 for [w_6_ + Pt]^2−^) were observed (Figure 3 and Figure 4a,c). These indicate that T_10_ was another platination site on ODN15, and the region from T_11_ to C_15_ was not platinated. Meanwhile, several either Pt-free or mono-platinated internal fragments [B_m_: B_n_] were also identified, accompanied by the observation of larger mono-platinated w_7_, w_10_, w_13_, and w_14_ fragments (Figure 3 and Figure 4a). However, neither the w nor a/[a − B] fragments were directly evident for the identification of the expected binding of cisplatin at G_8_, for which the major reason is attributed to the facile loss of guanine base during the CID fragmentation as previously reported by us [31]. Two short platinated internal fragments [T_6_: G_8_ + Pt(NH_3_)_2_]^−^ (obs. *m/z* 1341.14373 and calc. *m/z* 1341.14286) and [T_6_: G_8_ + Pt(NH_3_)]^−^ (obs. *m/z* 1324.11560 and calc. *m/z* 1324.11633) (Figure 3c, Figure 4a,d) were observed. This means that G_8_ may be the platination site, though it cannot exclude the binding of cisplatin at either T_6_ or T_7_. Collectively, the MS/MS data of mono-platinated ODN15 allowed us to unambiguously identify T_4_ and T_10_ as platination sites. Previous report also used FT-ICR MS to study the interactions of cisplatin with 13-mer double-stranded oligonucleotides, namely, GTATTGGCACGTA/TACGTGCCAATAC (ds1) and GTACCGGTGTGTA/TACACACCGGTAC (ds2); however, no thymine site was obtained [33]. This may be attributed to the competition of formation of purine crosslinks in the strand that prevented the formation of thymine-platinated adducts.

**Figure 5:**
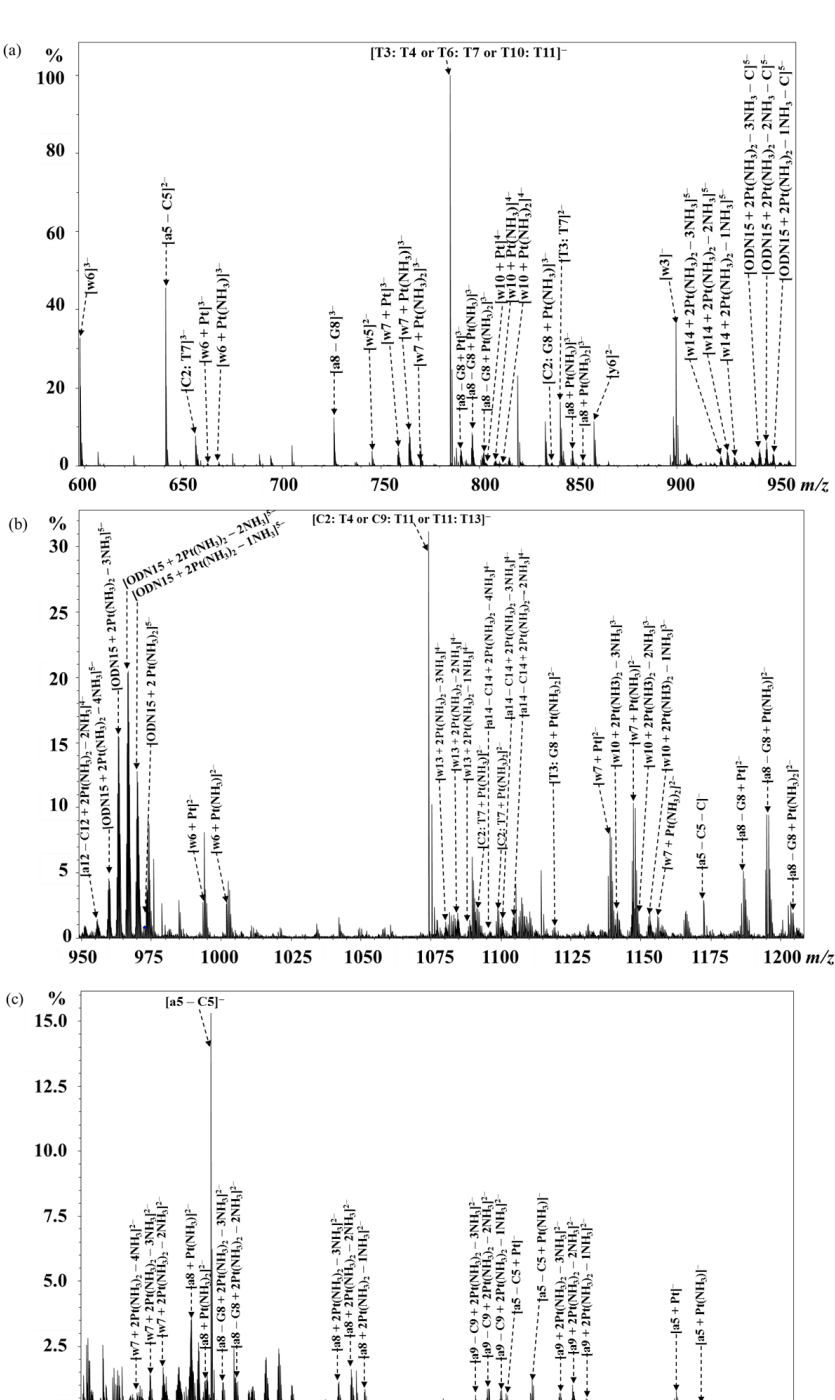
FT-ICR CID MS/MS spectra of [ODN15 + 2Pt(NH_3_)_2_ – 9H]^5−^ in the mass ranges *m/z* 600–950 (**a**), 950–1200 (**b**), and 1200–1650 (**c**). Main fragments containing platinum or platinum-free were assigned and labeled in the spectra.

**Figure 6:**
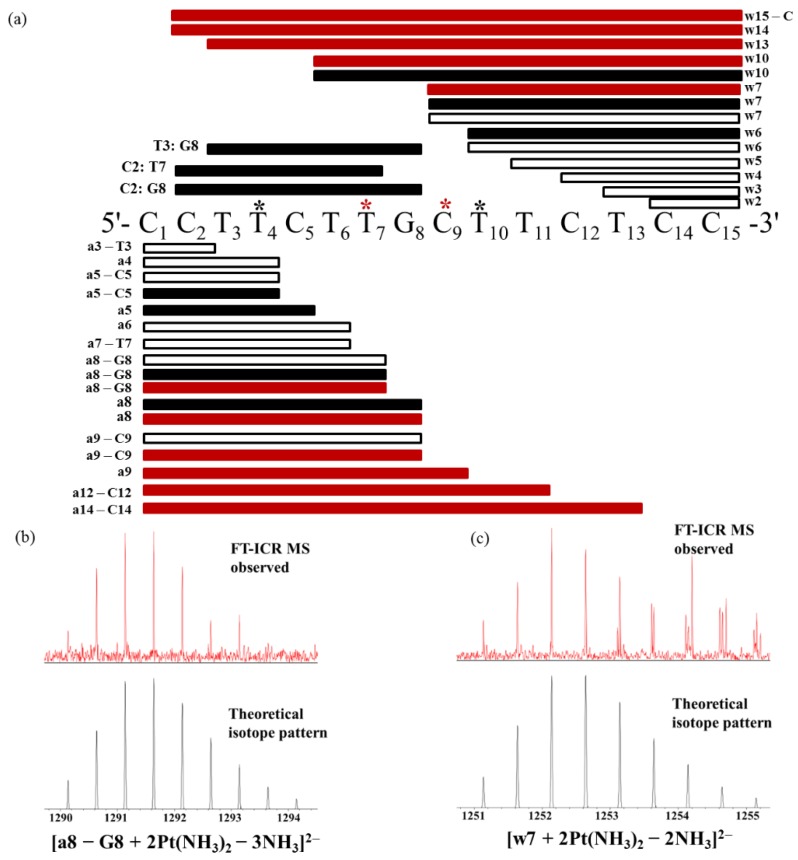
(**a**) Diagrammatic illustration of the localization of the binding sites for cisplatin on ODN15 based on the CID fragments of [ODN15 + 2 Pt(NH_3_)_2_ − 9H]^5−^ ion observed by MS/MS analysis. Blank box: platinum-free fragments; black-filled box: mono-platinated fragments; red-filled box: di-platinated fragments. * indicates the platination sites deduced by the mono-platinated fragments and * indicates those deduced by the di-platinated fragments. FT-ICR MS observed (**top**) and theoretical (**bottom**) isotope pattern of [a_8_ − G_8_ + 2Pt(NH_3_)_2_ − 3NH_3_]^2−^ (**b**) and [w_7_ + 2Pt(NH_3_)_2_ − 2NH_3_]^2−^ (**c**).

Further, the di-platinated ODN15 adduct [ODN15 + 2Pt(NH_3_)_2_ − 9H]^5−^ was fragmented by CID to identify the platination sites on it, and the results are shown in Figure 5 and Table S5. By applying a similar interpretation method described above, the T_4_ and T_10_ binding sites were deduced by the mono-platinated a and w fragments from 5′-terminal and 3′-terminal, respectively (Figure 5 and Figure 6a). Furthermore, from the 5′-terminal, no di-platinated a/[a − B] fragment was observed until [a_8_ − G_8_] (obs. *m/z* 1291.63534 and calc. *m/z* 1291.63593 for [a_8_ − G_8_ + 2Pt(NH_3_)_2_ − 3NH_3_]^2−^, Figure 5 and Figure 6a,b). This indicates that T_7_ was the second platination site apart from T_4_ on the di-platinated [a_8_ − G_8_] fragment. From the 3′-terminal, no di-platinated w fragment was observed until w_7_ (obs. *m/z* 1252.63603, calc. *m/z* 1252.63625 for [w_7_ + 2Pt(NH_3_)_2_ − 2NH_3_]^2−^, Figure 5 and Figure 6a,c), suggesting that C_9_ was another platination site besides T_10_ on the di-platinated w_7_ fragment.

Additionally, it is worthwhile to mention that the w fragment ions were sequential from w_1_ to w_14_ in the CID MS/MS spectrum of free ODN15 (Figure S1b,c) whereas they became intermittent in the fragmentation of mono-platinated and di-platinated ODN15, particularly for the later which lacked w_8_, w_9_, w_11_, and w_12_ fragments (Figure 4a and Figure 6a). The fragmentation sites of these w ions were located on both sides of T_7_ and T_4_, respectively, which were the binding sites for cisplatin on the di-platinated ODN15. This implies that the platination at T_7_ and T_4_ may retard the production of related w fragment ions, which can serve as a clue to help us locate the platination sites.

### 2.3. Binding Models of Cisplatin to Pyrimidines

**Figure 7:**
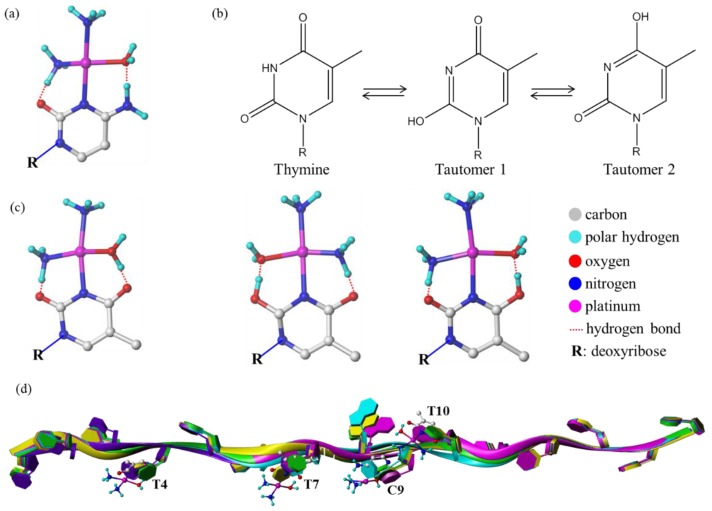
(**a**) Binding model of cisplatin with cytosine at N(3) site. (**b**) The tautomerization and tautomers of thymine. (**c**) Binding models of cisplatin with thymine and its tautomers at N(3) site. (**d**) Merged molecular models of ODN 15 (green), cisplatin-ODN15 adducts at T_4_ (purple), T_7_ (yellow), C_9_ (cyan), and T_10_ (magenta) site.

The well-established order of reactivity of the nucleotides with cisplatin was GMP > AMP >> CMP > UMP/TMP, indicating that pyrimidine nucleobases have low affinity to cisplatin [11], but the binding of Pt with pyrimidine derivatives have been widely confirmed [14,17,19]. In the present study, the binding of cisplatin to both thymine and cytosine on the oligodeoxynucleotide ODN15 was observed for the first time by FT-ICR MS. The exact coordination position of platinum on T and C was supposed to be the N3 atoms for both bases. It is well known that in duplex DNA at neutral pH, N3−H of T and C is H-bonded to adenine and guanine bases, respectively, and the N3 atom is inaccessible to metal ions. Thus, T and C are likely to be accessible only in single-stranded DNA, human telomere DNA, or the T or C base flipping out from the double helix when DNA is “breathing” [42,43,44]. The coordination of cisplatin to cytosine generally occurred at cytosine-N(3) position, which is a good nucleophilic site for the platinum electrophile [14,45,46]. The schematic diagram of binding model of cisplatin with cytosine was drawn using Sybyl X software and shown in Figure 7a. It has been previously demonstrated that the reaction of cisplatin with uracil and thymine is much slower than that with other nucleobases, especially in dilute solution (<0.1 mM), which accounts for the previous report that uracil and thymine did not react with cisplatin [14,46,47,48]. In our study, the concentration of ODN15 in the reaction solution with cisplatin was 0.5 mM and the corresponding concentration of thymine was up to 3.5 mM as there were 7 thymidine residues on ODN15. Moreover, the reaction was carried out at 310 K for 72 h. Therefore, high concentration combined with long reaction time increased the probability of cisplatin binding to thymine. Furthermore, the high sensitivity, high resolution, and mass accuracy of FT-ICR MS contribute to the detection and identification of platinated thymine and cytosine on ODN15.

The coordination of cisplatin to thymine was found to occur at thymine-N(3), with displacement of the proton or tautomerization of thymine [14,17,19,49]. The hydrogen bonded to N(3) is prone to depart due to the strong polarity of the N(3)-H bond induced by the adjacent two C=O bonds, making it possible for the displacement of proton by platinum. This was supported by the small decrease of the pH (~0.3) of the reaction solution of cisplatin and ODN15 with pH 5.92 at 0 h and 5.65 at 72 h. Moreover, the possible formation of intramolecular hydrogen bond from the aqua and ammonia ligands of cisplatin to the C(2) and C(4) carbonyl oxygen of thymine may also contribute to stabilizing the platinum-thymine adduct [14,50]. In a study reported by Kozelka et al. in 2005 [51], T-N3 was identified as the cisplatin binding site on a single-stranded dT20 oligonucleotide with no guanine in the sequence by using 2D [1H,15N] HMQC NMR spectroscopy. Such result was obtained at an acidic solution with a pH of 3.5 ± 0.5, where the competition from protonation at N3 was much stronger than that at a neutral solution similar to the present work. This result, together with our data present, demonstrate that cisplatin can compete with proton and guanine for binding to T-N3, implying that pyrimidine bases also play a role in the platination of DNA by cisplatin.

A previous report based on the computational work suggested that Pt^II^ initially binds to O4 and/or O2 of the canonical thymine tautomer and then migrates to N3 after its deprotonation [52]. However, in other reports based on experimental work, the N3,O4-crosslinked adduct between cisplatin and thymine was initially formed during the first two days and subsequently rearranged to N3,N3-crosslinks in a very slow reaction [51,53]. Therefore, the 72-h long incubation time used in the present work will most likely produce T-N3 bound adducts.

Another binding model of cisplatin with thymine is the tautomerization of thymine to make N(3) exposed and available for platinum coordination (Figure 7b), and the bound metal would promote this tautomerization, which is termed as metal-assisted thymine tautomerization [17,18]. The schematic diagram of binding models of cisplatin with thymine and thymine tautomers were drawn using Sybyl X software and shown in Figure 7c.

To study the structural consequence of ODN15 platination, we performed molecular modeling for ODN15 and cisplatin-ODN15 adducts with platination at T_4_, T_7_, C_9_, and T_10_. As shown in Figure 7d, the binding of cisplatin to ODN15 caused apparent distortion in the DNA strand, and the platination of different base sites on ODN15 led to different DNA structural alteration, which possibly initiates distinct downstream effects. However, elucidation of the exact binding models of cisplatin with the pyrimidine bases on DNA and the corresponding structural consequence still needs further investigation and integration with other techniques, such as NMR spectroscopy, X-ray diffraction analysis, etc.

## 3. Materials and Methods

### 3.1. Materials

Cisplatin was purchased from Beijing Ouhe Technology (Beijing, China). HPLC purified 15-mer oligodeoxynucleotide (5′-CCTTCTTGCTTCTCC-3′, ODN15) was obtained from Sangon Biotech. (Shanghai, China), and its concentration was determined by UV spectrometer (UV-2550, SHIMADZU, Nagoya, Japan) at 260 nm. Aqueous solutions were prepared using MilliQ water (Millipore, Milford, MA, USA).

### 3.2. Sample Preparation

It is well-established that cisplatin has the highest affinity to guanine. In order to make sure that the binding of cisplatin at bases other than the single guanine base in ODN15 was not due to the saturation of guanine by platinum, ODN15 (0.5 mM) reacted with equimolar amount of cisplatin in the dark at 310 K (mimic the body temperature) for 72 h to produce the platinated ODN15. The pH of the unbuffered reaction solution at 0 h and 72 h was measured to be 5.92 and 5.65, respectively. The reaction mixture was 7-fold diluted by water and then directly infused into ESI-FT-ICR MS (Bruker Daltonik GmbH, Bremen, Germany) for analysis.

### 3.3. FT-ICR Mass Spectrometry Analysis

All experiments were carried out on a SolariX 9.4T FT-ICR Mass Spectrometer (Bruker Daltonik GmbH, Bremen, Germany) under negative mode. For direct infusion ESI-FT-ICR MS experiments, the TD (Acquisition) was set to 1 M and ion accumulation time was 1.000 s. For collision-induced dissociation (CID) MS/MS experiments, the 5−charged precursor ions were isolated in quadrupole using an isolation window of 3–5 *m*/*z*. The collision energy was set to 14 V for free ODN15, 13–14 V for mono-platinated ODN15, and 14–15 V for di-platinated ODN15.

Bruker Compass Data Analysis v4.0 (Bruker Daltonik GmbH, Bremen, Germany) was used for data analysis and post processing. All spectra were internally calibrated using a quadratic calibration function and then interpreted and assigned manually with the help of the web-based Mongo Oligo Mass Calculator v2.08 (Hosted by The RNA Institute, College of Arts and Sciences, State University of New York at Albany, NY, USA, http://mods.rna.albany.edu/masspec/Mongo-Oligo). Additionally, theoretical and measured isotopic patterns for all assigned fragments were compared and their mass accuracy was calculated.

### 3.4. Molecular Modeling

The binding models of ODN15 with cisplatin were constructed using the Sybyl X software (ver. 1.1, Tripos Inc., El Cerrito, CA, USA). The ODN15 DNA strand model was built with Gasteiger–Huckel charges by the software and was energy-minimized using the Tripos force field with a distance-dependent dielectric function and Powell gradient algorithm with maximum iterations of 5000 and an energy convergence value of 0.05 kcal/mol. Cisplatin model was created and manually adjusted to coordinate to cytidine-(N3) or deprotonated thymine-(N3) on ODN15. Then, Gasteiger–Huckel charges were added to the cisplatin-ODN15 adduct followed by the energy-minimization using Tripos force field with the addition of parameter file specifically for metal prior to the minimization.

## 4. Conclusions

Cisplatin exerts its anticancer activity ultimately through covalent binding to DNA, causing deformation of the DNA structure by crosslinking neighbouring bases and eventually leading to cancer cell apoptosis. The binding sites of cisplatin to DNA and the resulting structural alteration of DNA play a critical role in the mechanism of action of cisplatin. In this study, the unexpected thermodynamic binding of cisplatin to thymine and cytosine on a 15-mer C,T-rich oligodeoxynucleotide (ODN15) was firstly identified by high resolution FT-ICR MS with CID fragmentation. The abundant and informative Pt-containing or Pt-free w, a/[a − B] and internal fragments allow the unambiguous identification of Pt binding to T_4_, T_7_, C_9_, and T_10_ on ODN15. To the best of our knowledge, this is the first observation of the binding of cisplatin with thymine and cytosine bases at the oligonucleotide level using FT-ICR MS, which demonstrates the great potential and superiority of FT-ICR MS in studying the interactions of metallodrugs with large biomolecules (e.g., DNA, RNA, and proteins). Further investigations using other techniques are still required to clarify the exact binding models of cisplatin with pyrimidine bases on DNA and the precise structure of the Pt-DNA adducts. Recently, the preference and selectivity for gene loci of cisplatin binding to cellular genome DNA has attracted increasing attention [54]. We anticipate that our findings could provide new insights into the gene preference and the diversity of cisplatin binding.

## Supplementary Materials

The following are available online at https://www.mdpi.com/1420-3049/24/10/1852/s1, Tables S1 and S2: The full MS peak assignments of ODN15 (Table S1) and the reaction mixture of ODN15 with cisplatin (Table S2); Tables S3–S5: The CID MS/MS fragments assignment of [ODN15 − 5H]^5−^ (Table S3), [ODN15 + Pt(NH_3_)_2_ − 7H]5^−^ (Table S4) and [ODN15 + 2Pt(NH_3_)_2_ − 9H]^5−^ (Table S5); Figure S1: CID MS/MS spectrum of [ODN15 − 5H]^5−^, along with corresponding fragmentation maps and fragments coverage of ODN15.
