# Supplementary material for: Discovery of Cisplatin Binding to Thymine and Cytosine on a Single-Stranded Oligodeoxynucleotide by High Resolution FT-ICR Mass Spectrometry
Source: Molecules. 2019 May 14;24(10):1852. doi: 10.3390/molecules24101852 (PMC6571787; doi:10.3390/molecules24101852)
Supplement: Supplementary file 1 [file molecules-24-01852-s001.pdf]

Discovery of Cisplatin Binding to Thymine and Cytosine on a Single-stranded Oligodeoxynucleotide by High Resolution FT-ICR Mass Spectrometry

Wenjuan Zeng[1,3], Yanyan Zhang[1], Wei Zheng[1], Qun Luo[1,3], Juanjuan Han[1], Jian'an Liu[1], Yao Zhao[1], Feifei Jia[1], Kui Wu[2,*] and Fuyi Wang[1,3,4,*]

[1] Beijing National Laboratory for Molecular Sciences; National Centre for Mass Spectrometry in Beijing; CAS Key Laboratory of Analytical Chemistry for Living Biosystems, Institute of Chemistry, Chinese Academy of Sciences, Beijing 100190, P. R. China

[2] Key Laboratory of Hubei Province for Coal Conversion and New Carbon Materials; School of Chemistry and Chemical Engineering, Wuhan University of Science and Technology , Wuhan, 430081, P. R. China

[3] University of Chinese Academy of Sciences, Beijing 100049, P. R. China

[4] Basic Medical College, Shandong University of Chinese Traditional Medicine, Jinan 250355, P. R. China

Table S1. Full MS peak assignments of ODN15 for Figure 1a. Red-highlighted species were used for the internal calibration.

5′ HO-CCTTCTTGCTTCTCC-OH 3′ (ODN15, $C_{143}H_{188}N_{40}O_{95}P_{14}$)	Observed m/z	Exact m/z	Mass error/ ppm
$[ODN15 - 3H]^{3-}$	1472.24136	1472.241555	–0.13
$[ODN15 - 4H]^{4-}$	1103.92967	1103.929347	0.29
$[ODN15 - 5H]^{5-}$	882.94202	882.942022	0.00
$[ODN15 - 6H]^{6-}$	735.61733	735.617139	0.26

Table S2. Full MS peak assignments of the reaction mixture of ODN15 with cisplatin for Figure 1b. Red-highlighted species were used for the internal calibration.

	Observed m/z	Exact m/z	Mass error/ ppm
$[ODN15 + 2Pt(NH_3)_2 - 7H]^{3-}$	1623.90939	1623.910057	–0.41
$[ODN15 + 1Pt(NH_3)_2 - 5H]^{3-}$	1548.24333	1548.242972	0.23
$[ODN15 - 3H]^{3-}$	1472.24262	1472.241555	0.72
$[ODN15 + 2Pt(NH_3)_2 - 8H]^{4-}$	1217.68075	1217.680724	0.02
$[ODN15 + 1Pt(NH_3)_2 - 6H]^{4-}$	1160.92891	1160.930410	–1.29
$[ODN15 - 4H]^{4-}$	1103.93047	1103.929347	1.02
$[ODN15 + 2Pt(NH_3)_2 - 9H]^{5-}$	973.94292	973.943124	–0.21
$[ODN15 + 1Pt(NH_3)_2 -7H]^{5-}$	928.54282	928.542873	–0.06
$[ODN15 - 5H]^{5-}$	882.94279	882.942022	0.87
$[ODN15 + 1Pt(NH_3)_2 -8H]^{6-}$	773.61994	773.617848	2.70
$[ODN15 - 6H]^{6-}$	735.61969	735.617139	3.46

Table S3. CID MS/MS fragments assignment of $[ODN15 - 5H]^{5-}$ for Figure S1a. Red-highlighted species were used for the internal calibration.

Fragment	Observed m/z	Exact m/z	Mass error/ ppm	Chemical formula of neutral fragment
$[w1]^{-}$	306.04966	306.049660	0.00	C9H14N3O7P
$[w2]^{-}$	595.09650	595.096032	0.79	C18H26N6O13P2
$[w3]^{-}$	899.14180	899.142069	–0.30	C28H39N8O20P3
$[w3]^{2-}$	449.06751	449.067397	0.25	C28H39N8O20P3
$[w4]^{-}$	1188.18895	1188.18844	0.43	C37H51N11O26P4
$[w4]^{2-}$	593.59108	593.590583	0.84	C37H51N11O26P4
$[w5]^{2-}$	745.61421	745.613601	0.82	C47H64N13O33P5
$[w6]^{2-}$	897.63694	897.636620	0.36	C57H77N15O40P6
$[w6]^{3-}$	598.08880	598.088654	0.24	C57H77N15O40P6
$[w7]^{2-}$	1042.15903	1042.15981	–0.74	C66H89N18O46P7
$[w7]^{3-}$	694.43766	694.437445	0.31	C66H89N18O46P7
$[w8]^{3-}$	804.12195	804.121618	0.41	C76H101N23O52P8
$[w9]^{4-}$	678.85154	678.850904	0.94	C86H114N25O59P9

[w9]$^{3-}$	905.46996	905.470297	–0.37	C86H114N25O59P9
[w10]$^{4-}$	754.86246	754.862413	0.06	C96H127N27O66P10
[w11]$^{4-}$	827.12432	827.124006	0.38	C105H139N30O72P11
[w12]$^{4-}$	903.13541	903.135516	–0.12	C115H152N32O79P12
[w13]$^{4-}$	979.14746	979.147025	0.44	C125H165N34O86P13
[w13]$^{5-}$	783.11687	783.116165	0.90	C125H165N34O86P13
[a2 – C5]$^{-}$	386.07598	386.075875	0.27	C14H18N3O8P
[a3 – T3]$^{-}$	675.12276	675.122247	0.76	C23H30N6O14P2
[a4]$^{-}$	1105.21176	1105.211210	0.50	C38H49N10O23P3
[a5 – C5]$^{-}$	1283.21464	1283.214320	0.25	C43H56N10O28P4
[a5 – C5]$^{2-}$	641.10360	641.103523	0.12	C43H56N10O28P4
[a5]$^{-}$	1394.25907	1394.257580	1.07	C47H61N13O29P4
[a6]$^{2-}$	848.64911	848.648172	1.10	C57H74N15O36P5
[a7 – T7]$^{2-}$	937.64974	937.649727	0.01	C62H81N15O41P6
[a8 – G8]$^{3-}$	726.11292	726.112739	0.25	C72H94N17O48P7
[a8 – G8]$^{2-}$	1089.67270	1089.672750	–0.04	C72H94N17O48P7
[a8]$^{2-}$	1165.19639	1165.197450	–0.91	C77H99N22O49P7
[a9 – C9]$^{3-}$	835.79744	835.796912	0.63	C82H106N22O54P8
[a9 – C9]$^{2-}$	1254.19810	1254.199010	–0.72	C82H106N22O54P8
[a12 – C12]$^{3-}$	1134.84408	1134.843060	0.90	C111H144N29O74P11
[a12 – C12]$^{4-}$	850.88101	850.880476	0.63	C111H144N29O74P11
[a14 – C12]$^{4-}$	999.15333	999.153579	–0.25	C130H169N34O87P13
[T3: T4 or T6: T7 or T10: T11]$^{-}$	785.08769	785.087908	–0.28	C25H33N4O19P3
[T6: G8]$^{2-}$	556.56698	556.566576	0.72	C35H45N9O25P4
[T6: G8]$^{-}$	1114.14052	1114.140430	0.08	C35H45N9O25P4
[C2: T7]$^{3-}$	656.41951	656.419391	0.18	C63H83N14O45P7
[C2: T7]$^{2-}$	985.13319	985.132725	0.47	C63H83N14O45P7
[T3: T7]$^{2-}$	840.60936	840.609540	–0.21	C54H71N11O39P6
[T3: T7]$^{-}$	1682.22732	1682.226360	0.57	C54H71N11O39P6
[C2: T6 or C9: T13 or T10: C14]$^{2-}$	833.10982	833.109707	0.14	C53H70N12O38P6
[C2: T6 or C9: T13 or T10: C14]$^{-}$	1667.22835	1667.226690	1.00	C53H70N12O38P6

Table S4. CID MS/MS fragments assignment of [ODN15 + $Pt(NH_3)_2$ − 7H]$^{5-}$ for Figure 3. Red-highlighted species were used for the internal calibration.

Fragment	Observed m/z	Exact m/z	Mass error/ ppm	Chemical formula of neutral fragment
[w7 + Pt]$^{3-}$	759.08848	759.087344	1.49	C66H87N18O46P7Pt
[w7 + $Pt(NH_3)$]$^{3-}$	764.76398	764.762859	1.46	C66H90N19O46P7Pt
[w7 + $Pt(NH_3)_2$]$^{3-}$	770.43957	770.4383743	1.55	C66H93N20O46P7Pt

$[w7 + Pt]^{2-}$	1139.1367	1139.134654	1.79	C66H87N18O46P7Pt
$[w7 + Pt(NH_3)]^{2-}$	1147.64902	1147.647927	0.95	C66H90N19O46P7Pt
$[w7 + Pt(NH_3)_2]^{2-}$	1156.16248	1156.1612	1.11	C66H93N20O46P7Pt
$[a8 – G8 + Pt]^{3-}$	790.76357	790.7634657	0.13	C72H92N17O48P7Pt
$[a8 – G8 + Pt(NH_3)]^{3-}$	796.43932	796.438981	0.43	C72H95N18O48P7Pt
$[a8 – G8 + Pt(NH_3)_2]^{3-}$	802.11504	802.1144963	0.68	C72H98N19O48P7Pt
$[a8 – G8 + Pt]^{2-}$	1186.64877	1186.648837	–0.06	C72H92N17O48P7Pt
$[a8 – G8 + Pt(NH_3)]^{2-}$	1195.16194	1195.16211	–0.14	C72H95N18O48P7Pt
$[a8 – G8 + Pt(NH_3)_2]^{2-}$	1203.67438	1203.675383	–0.83	C72H98N19O48P7Pt
$[w10 + Pt]^{4-}$	803.35116	803.350266	1.11	C96H125N27O66P10Pt
$[w10 + Pt(NH_3)]^{4-}$	807.60749	807.6069	0.73	C96H128N28O66P10Pt
$[w10 + Pt(NH_3)_2]^{4-}$	811.8643	811.863534	0.94	C96H131N29O66P10Pt
$[w10 + Pt]^{3-}$	1071.46945	1071.469447	0.00	C96H125N27O66P10Pt
$[w10 + Pt(NH_3)]^{3-}$	1077.14567	1077.144959	0.66	C96H128N28O66P10Pt
$[w10 + Pt(NH_3)_2]^{3-}$	1082.82142	1082.820471	0.88	C96H131N29O66P10Pt
$[w13 + Pt(NH_3)]^{5-}$	825.31257	825.311805	0.93	C125H166N35O86P13Pt
$[w13 + Pt(NH_3)_2]^{5-}$	828.71791	828.7171124	0.96	C125H169N36O86P13Pt
$[w13 + Pt]^{4-}$	1027.63449	1027.634941	–0.44	C125H163N34O86P13Pt
$[w13 + Pt(NH_3)]^{4-}$	1031.89221	1031.891575	0.61	C125H166N35O86P13Pt
$[w13 + Pt(NH_3)_2]^{4-}$	1036.1491	1036.14821	0.86	C125H169N36O86P13Pt
$[a8 + Pt]^{3-}$	840.77967	840.77904	0.75	C77H97N22O49P7Pt
$[a8 + Pt(NH_3)]^{3-}$	846.78947	846.7887857	0.81	C77H100N23O49P7Pt
$[a8 + Pt(NH_3)_2]^{3-}$	852.46488	852.464301	0.68	C77H103N24O49P7Pt
$[a8 + Pt(NH_3)]^{2-}$	1270.68536	1270.686817	–1.15	C77H100N23O49P7Pt
$[a8 + Pt(NH_3)_2]^{2-}$	1279.19885	1279.20009	–0.97	C77H103N24O49P7Pt
$[w14 + Pt]^{5-}$	879.71657	879.7157826	0.89	C134H175N37O92P14Pt
$[w14 + Pt(NH_3)]^{5-}$	883.12161	883.1210902	0.59	C134H178N38O92P14Pt
$[w14 + Pt(NH_3)_2]^{5-}$	886.52753	886.5263976	1.28	C134H181N39O92P14Pt
$[ODN15 + Pt(NH_3) – G8]^{5-}$	894.72829	894.7272292	1.18	C138H184N36O94P14Pt
$[ODN15 + Pt – C]^{5-}$	899.32379	899.323148	0.71	C139H181N37O94P14Pt
$[ODN15 + Pt(NH_3) – C]^{5-}$	902.72903	902.7284556	0.64	C139H184N38O94P14Pt
$[ODN15 + Pt(NH_3)_2 – C]^{5-}$	906.13434	906.1337632	0.64	C139H187N39O94P14Pt
$[ODN15 + Pt]^{5-}$	921.73289	921.7322602	0.68	C143H186N40O95P14Pt
$[ODN15 + Pt(NH_3)]^{5-}$	925.13772	925.1375664	0.17	C143H189N41O95P14Pt
$[ODN15 + Pt(NH_3)_2]^{5-}$	928.54361	928.5428726	0.79	C143H192N42O95P14Pt
$[a9 – C9 + Pt(NH_3)]^{3-}$	906.12358	906.1231627	0.46	C82H107N23O54P8Pt
$[a9 – C9 + Pt(NH_3)_2]^{3-}$	911.79942	911.7986783	0.81	C82H110N24O54P8Pt
$[a9 – C9 + Pt]^{2-}$	1351.17528	1351.175109	0.13	C82H104N22O54P8Pt
$[a9 – C9 + Pt(NH_3)]^{2-}$	1359.68805	1359.688382	–0.24	C82H107N23O54P8Pt
$[a9 – C9 + Pt(NH_3)_2]^{2-}$	1368.20063	1368.201656	–0.75	C82H110N24O54P8Pt
$[a9 + Pt]^{3-}$	937.46244	937.46207	0.39	C86H109N25O55P8Pt
$[a9 + Pt(NH_3)]^{3-}$	943.13757	943.1375857	–0.02	C86H112N26O55P8Pt
$[a9 + Pt(NH_3)_2]^{3-}$	948.81376	948.813101	0.69	C86H115N27O55P8Pt

[w6 + Pt]$^{2-}$	994.61317	994.611457	1.72	C57H75N15O40P6Pt
[w6 + Pt(NH$_3$)]$^{2-}$	1003.12584	1003.12473	1.11	C57H78N16O40P6Pt
[a14 – C14 + Pt]$^{4-}$	1047.64331	1047.641507	1.72	C130H167N34O87P13Pt
[a14 – C14 + Pt(NH$_3$)]$^{4-}$	1051.89936	1051.898142	1.16	C130H170N35O87P13Pt
[a14 – C14 + Pt(NH$_3$)$_2$]$^{4-}$	1056.15637	1056.154776	1.51	C130H173N36O87P13Pt
[a12 – C12 + Pt]$^{3-}$	1199.49403	1199.493584	0.37	C111H142N29O74P11Pt
[a12 – C12 + Pt(NH$_3$)]$^{3-}$	1205.16828	1205.169097	–0.68	C111H145N30O74P11Pt
[a12 – C12 + Pt(NH$_3$)$_2$]$^{3-}$	1210.84412	1210.844609	–0.40	C111H148N31O74P11Pt
[a12 – C12 + Pt(NH$_3$)]$^{4-}$	903.62564	903.6250035	0.70	C111H145N30O74P11Pt
[a12 – C12 + Pt(NH$_3$)$_2$]$^{4-}$	907.88211	907.8816375	0.52	C111H148N31O74P11Pt
[T6: G8 + Pt(NH$_3$)]$^{-}$	1324.1156	1324.116325	–0.55	C35H46N10O25P4Pt
[T6: G8 + Pt(NH$_3$)$_2$]$^{-}$	1341.14373	1341.142861	0.65	C35H49N11O25P4Pt
[a5 – C5 + Pt]$^{-}$	1476.16467	1476.16373	0.64	C43H54N10O28P4Pt
[a5 – C5 + Pt(NH$_3$)]$^{-}$	1493.19076	1493.190267	0.33	C43H57N11O28P4Pt
[a5 – C5 + Pt(NH$_3$)$_2$]$^{-}$	1510.21711	1510.216804	0.20	C43H60N12O28P4Pt
[a5 + Pt]$^{-}$	1587.20856	1587.206982	0.99	C47H59N13O29P4Pt
[a5 + Pt(NH$_3$)]$^{-}$	1604.23226	1604.23352	–0.78	C47H62N14O29P4Pt
[w14 – C + Pt(NH$_3$)]$^{5-}$	860.91327	860.9124374	0.97	C130H173N35O91P14Pt
[w14 – C + Pt(NH$_3$)$_2$]$^{5-}$	864.31809	864.3177448	0.40	C130H176N36O91P14Pt
[a9 – C9 – C + Pt]$^{2-}$	1295.6523	1295.653475	–0.91	C78H99N19O53P8Pt
[a9 –C9 – C + Pt(NH$_3$)]$^{2-}$	1304.16614	1304.166748	–0.47	C78H102N20O53P8Pt
[a9 – C9 – C + Pt(NH$_3$)$_2$]$^{2-}$	1312.67988	1312.680022	–0.11	C78H105N21O53P8Pt
[C2: G8 + Pt(NH$_3$)]$^{3-}$	836.43043	836.4298067	0.74	C73H96N20O51P8Pt
[C2: G8 + Pt]$^{2-}$	1246.63411	1246.635075	–0.77	C73H93N19O51P8Pt
[C2: G8 + Pt(NH$_3$)]$^{2-}$	1255.14711	1255.148348	–0.99	C73H96N20O51P8Pt
[C2: G8 + Pt(NH$_3$)$_2$]$^{2-}$	1263.66057	1263.661622	–0.83	C73H99N21O51P8Pt
[C2: T7 + Pt]$^{2-}$	1082.10928	1082.107576	1.57	C63H81N14O45P7Pt
[C2: T7 + Pt(NH$_3$)]$^{2-}$	1090.62237	1090.620849	1.39	C63H84N15O45P7Pt
[C2: T7 + Pt(NH$_3$)$_2$]$^{2-}$	1099.13529	1099.134122	1.06	C63H87N16O45P7Pt
[T3: G8 + Pt]$^{2-}$	1102.11104	1102.110648	0.36	C64H81N16O45P7Pt
[T3: G8 + Pt(NH$_3$)]$^{2-}$	1110.62522	1110.623921	1.17	C64H84N17O45P7Pt
[T3: G8 +Pt(NH$_3$)$_2$]$^{2-}$	1119.13879	1119.137193	1.43	C64H87N18O45P7Pt
[C2: T11 + Pt]$^{3-}$	1129.80041	1129.800215	0.17	C102H131N26O71P11Pt
[C2: T11 + Pt(NH$_3$)]$^{3-}$	1135.47679	1135.475727	0.94	C102H134N27O71P11Pt
[w2]$^{-}$	595.09644	595.096032	0.68	C18H26N6O13P2
[w3]$^{-}$	899.14198	899.142069	–0.10	C28H39N8O20P3
[w3]$^{2-}$	449.0674	449.0673965	0.01	C28H39N8O20P3
[w4]$^{-}$	1188.19246	1188.188441	3.38	C37H51N11O26P4
[w4]$^{2-}$	593.59109	593.5905825	0.85	C37H51N11O26P4
[w5]$^{2-}$	745.61429	745.613601	0.92	C47H64N13O33P5
[w6]$^{2-}$	897.63717	897.6366195	0.61	C57H77N15O40P6
[w6]$^{3-}$	598.0888	598.0886543	0.24	C57H77N15O40P6
[w7]$^{2-}$	1042.15992	1042.159806	0.11	C66H89N18O46P7

$[w7]^{3-}$	694.43799	694.437445	0.78	C66H89N18O46P7
$[w8]^{3-}$	804.12222	804.1216183	0.75	C76H101N23O52P8
$[w10]^{4-}$	754.86278	754.8624133	0.49	C96H127N27O66P10
$[a3 - T3]^{-}$	675.12285	675.122247	0.89	C23H30N6O14P2
$[a5 - C5]^{-}$	1283.21455	1283.214321	0.18	C43H56N10O28P4
$[a5 - C5]^{2-}$	641.10372	641.1035225	0.31	C43H56N10O28P4
$[a7 - T7]^{2-}$	937.65014	937.649727	0.44	C62H81N15O41P6
$[a8 - G8]^{3-}$	726.11321	726.1127387	0.65	C72H94N17O48P7
$[a8 - G8]^{2-}$	1089.67292	1089.672746	0.16	C72H94N17O48P7
$[a9 - C9]^{3-}$	835.79775	835.7969117	1.00	C82H106N22O54P8
$[a9 - C9]^{2-}$	1254.19796	1254.199006	–0.83	C82H106N22O54P8
$[a12 - C12]^{4-}$	850.88123	850.8804763	0.88	C111H144N29O74P11
$[T3: T4 \text{ or } T6: T7 \text{ or } T10: T11]^{-}$	785.08774	785.087908	–0.21	C25H33N4O19P3
$[C2: T6]^{2-}$	833.11013	833.1097065	0.51	C53H70N12O38P6
$[T3: T7]^{2-}$	840.60979	840.6095395	0.30	C54H71N11O39P6
$[a6]^{2-}$	848.64886	848.648172	0.81	C57H74N15O36P5
$[y6]^{2-}$	857.65402	857.6534545	0.66	C57H76N15O37P5
$[C2: T7]^{3-}$	656.41981	656.4193913	0.64	C63H83N14O45P7
$[C2: T7]^{2-}$	985.13372	985.132725	1.01	C63H83N14O45P7
$[y7]^{2-}$	1002.17713	1002.176641	0.49	C66H88N18O43P6
$[a4]^{-}$	1105.21191	1105.211211	0.63	C38H49N10O23P3
$[C2: T4 \text{ or } C9: T11 \text{ or } T11: T13]^{-}$	1074.13391	1074.13428	–0.34	C34H45N7O25P4
$[C2: T4 \text{ or } C9: T11 \text{ or } T11: T13]^{2-}$	536.56389	536.563502	0.72	C34H45N7O25P4
$[T3: T6 \text{ or } T4: T7 \text{ or } T10: T13]^{2-}$	688.58713	688.5865205	0.88	C44H58N9O32P5
$[T3: T6 \text{ or } T4: T7 \text{ or } T10: T13]^{-}$	1378.17878	1378.180317	–1.11	C44H58N9O32P5
$[a5]^{-}$	1394.25756	1394.257583	–0.02	C47H61N13O29P4
$[y3]^{-}$	819.1764	819.175739	0.81	C28H38N8O17P2

Table S5. CID MS/MS fragments assignment of $[ODN15 + 2Pt(NH_3)_2 - 9H]^{5-}$ for Figure 5. Red-highlighted species were used for the internal calibration.

Fragment	Observed m/z	Exact m/z	Mass error/ ppm	Chemical formula of neutral fragment
$[w6 + Pt]^{3-}$	662.73951	662.738546	1.45	C57H75N15O40P6Pt
$[w6 + Pt(NH_3)]^{3-}$	668.41467	668.4140613	0.91	C57H78N16O40P6Pt
$[w6 + Pt]^{2-}$	994.61232	994.611457	0.87	C57H75N15O40P6Pt
$[w6 + Pt(NH_3)]^{2-}$	1003.1251	1003.12473	0.37	C57H78N16O40P6Pt

$[w7 + Pt]^{3-}$	759.08836	759.087344	1.34	C66H87N18O46P7Pt
$[w7 + Pt(NH_3)]^{3-}$	764.76361	764.762859	0.98	C66H90N19O46P7Pt
$[w7 + Pt(NH_3)_2]^{3-}$	770.43911	770.4383743	0.95	C66H93N20O46P7Pt
$[w7 + Pt]^{2-}$	1139.13496	1139.134654	0.27	C66H87N18O46P7Pt
$[w7 + Pt(NH_3)]^{2-}$	1147.64834	1147.647927	0.36	C66H90N19O46P7Pt
$[w7 + Pt(NH_3)_2]^{2-}$	1156.16167	1156.161200	0.41	C66H93N20O46P7Pt
$[a8 - G8 + Pt]^{3-}$	790.76328	790.7634657	−0.23	C72H92N17O48P7Pt
$[a8 - G8 + Pt(NH_3)]^{3-}$	796.43894	796.438981	−0.05	C72H95N18O48P7Pt
$[a8 - G8 + Pt(NH_3)_2]^{3-}$	802.11458	802.1144963	0.10	C72H98N19O48P7Pt
$[a8 - G8 + Pt]^{2-}$	1186.64828	1186.648837	−0.47	C72H92N17O48P7Pt
$[a8 - G8 + Pt(NH_3)]^{2-}$	1195.16139	1195.16211	−0.60	C72H95N18O48P7Pt
$[a8 - G8 + Pt(NH_3)_2]^{2-}$	1203.67385	1203.675383	−1.27	C72H98N19O48P7Pt
$[w10 + Pt]^{4-}$	803.35077	803.350266	0.63	C96H125N27O66P10Pt
$[w10 + Pt(NH_3)]^{4-}$	807.60724	807.6069	0.42	C96H128N28O66P10Pt
$[w10 + Pt(NH_3)_2]^{4-}$	811.86409	811.863534	0.68	C96H131N29O66P10Pt
$[a8 + Pt(NH_3)]^{3-}$	846.78905	846.7887857	0.31	C77H100N23O49P7Pt
$[a8 + Pt(NH_3)_2]^{3-}$	852.46456	852.464301	0.30	C77H103N24O49P7Pt
$[a8 + Pt(NH_3)]^{2-}$	1270.68533	1270.686817	−1.17	C77H100N23O49P7Pt
$[a8 + Pt(NH_3)_2]^{2-}$	1279.19838	1279.20009	−1.34	C77H103N24O49P7Pt
$[a5 - C5 + Pt]^{2-}$	737.57866	737.578227	0.59	C43H54N10O28P4Pt
$[a5 - C5 + Pt]^{-}$	1476.16366	1476.16373	−0.05	C43H54N10O28P4Pt
$[a5 - C5 + Pt(NH_3)]^{-}$	1493.1902	1493.190267	−0.04	C43H57N11O28P4Pt
$[a5 + Pt]^{-}$	1587.20792	1587.206982	0.59	C47H59N13O29P4Pt
$[a5 + Pt(NH_3)]^{-}$	1604.23204	1604.23352	−0.92	C47H62N14O29P4Pt
$[C2{:}\ G8 + Pt(NH_3)]^{3-}$	836.42961	836.4298067	−0.23	C73H96N20O51P8Pt
$[C2{:}\ T7 + Pt]^{2-}$	1082.10797	1082.107576	0.36	C63H81N14O45P7Pt
$[C2{:}\ T7 + Pt(NH_3)]^{2-}$	1090.6218	1090.620849	0.87	C63H84N15O45P7Pt
$[C2{:}\ T7 + Pt(NH_3)_2]^{2-}$	1099.13378	1099.134122	−0.31	C63H87N16O45P7Pt
$[T3{:}\ G8 + Pt]^{2-}$	1102.11075	1102.110648	0.09	C64H81N16O45P7Pt
$[T3{:}\ G8 + Pt(NH_3)]^{2-}$	1110.62419	1110.623921	0.24	C64H84N17O45P7Pt
$[T3{:}\ G8 + Pt(NH_3)_2]^{2-}$	1119.1376	1119.137193	0.36	C64H87N18O45P7Pt
$[w14 + 2Pt(NH_3)_2 - 4NH_3]^{5-}$	918.50657	918.5058694	0.76	C134H173N37O92P14Pt2
$[w14 + 2Pt(NH_3)_2 - 3NH_3]^{5-}$	921.91135	921.9111762	0.19	C134H176N38O92P14Pt2
$[w14 + 2Pt(NH_3)_2 - 2NH_3]^{5-}$	925.31668	925.3164828	0.21	C134H179N39O92P14Pt2
$[w14 + 2Pt(NH_3)_2 - 1NH_3]^{5-}$	928.72235	928.7217894	0.60	C134H182N40O92P14Pt2
$[a12 - C12 + 2Pt(NH_3)_2 - 3NH_3]^{4-}$	951.86229	951.8621293	0.17	C111H143N30O74P11Pt2
$[a12 - C12 + 2Pt(NH_3)_2 - 2NH_3]^{4-}$	956.1192	956.118763	0.46	C111H146N31O74P11Pt2
$[ODN15 + 2Pt(NH_3)_2 - G8 - 3NH_3]^{5-}$	933.51768	933.5173226	0.38	C138H182N36O94P14Pt2
$[ODN15 + 2Pt(NH_3)_2 - G8 - 2NH_3]^{5-}$	936.92253	936.9226292	−0.11	C138H185N37O94P14Pt2

$[ODN15 + 2Pt(NH_3)_2 − 4NH_3]^{5−}$	960.32223	960.3218964	0.35	C143H184N40O95P14Pt2
$[ODN15 + 2Pt(NH_3)_2 − 3NH_3]^{5−}$	963.72756	963.7272032	0.37	C143H187N41O95P14Pt2
$[ODN15 + 2Pt(NH_3)_2 − 2NH_3]^{5−}$	967.13273	967.13251	0.23	C143H190N42O95P14Pt2
$[ODN15 + 2Pt(NH_3)_2 − 1NH_3]^{5−}$	970.53788	970.5378168	0.07	C143H193N43O95P14Pt2
$[ODN15 + 2Pt(NH_3)_2]^{5−}$	973.94306	973.9431236	–0.07	C143H196N44O95P14Pt2
$[w13 + 2Pt(NH_3)_2 − 3NH_3]^{4−}$	1080.12812	1080.128708	–0.54	C125H164N35O86P13Pt2
$[w13 + 2Pt(NH_3)_2 − 2NH_3]^{4−}$	1084.38475	1084.385342	–0.55	C125H167N36O86P13Pt2
$[w13 + 2Pt(NH_3)_2 − 1NH_3]^{4−}$	1088.64156	1088.641977	–0.38	C125H170N37O86P13Pt2
$[a14 − C14 + 2Pt(NH_3)_2 − 4NH_3]^{4−}$	1096.12968	1096.129111	0.52	C130H165N34O87P13Pt2
$[a14 − C14 + 2Pt(NH_3)_2 − 3NH_3]^{4−}$	1100.38611	1100.385745	0.33	C130H168N35O87P13Pt2
$[a14 − C14 + 2Pt(NH_3)_2 − 2NH_3]^{4−}$	1104.6428	1104.642378	0.38	C130H171N36O87P13Pt2
$[w10 + 2Pt(NH_3)_2 − 3NH_3]^{3−}$	1141.46129	1141.461118	0.15	C96H126N28O66P10Pt2
$[w10 + 2Pt(NH_3)_2 − 2NH_3]^{3−}$	1147.13523	1147.13663	–1.22	C96H129N29O66P10Pt2
$[w10 + 2Pt(NH_3)_2 − 1NH_3]^{3−}$	1152.81224	1152.812141	0.09	C96H132N30O66P10Pt2
$[w7 + 2Pt(NH_3)_2 − 4NH_3]^{2−}$	1235.10838	1235.108645	-0.21	C66H85N18O46P7Pt2
$[w7 + 2Pt(NH_3)_2 − 3NH_3]^{2−}$	1244.12297	1244.122978	–0.01	C66H88N19O46P7Pt2
$[w7 + 2Pt(NH_3)_2 − 2NH_3]^{2−}$	1252.63603	1252.636251	–0.18	C66H91N20O46P7Pt2
$[a8 − G8 + 2Pt(NH_3)_2 − 3NH_3]^{2−}$	1291.63534	1291.635929	–0.46	C72H93N18O48P7Pt2
$[a8 − G8 + 2Pt(NH_3)_2 − 2NH_3]^{2−}$	1300.14868	1300.149202	–0.40	C72H96N19O48P7Pt2
$[a8 − G8 + 2Pt(NH_3)_2 − 1NH_3]^{2−}$	1308.66248	1308.662379	0.08	C72H99N20O48P7Pt2
$[a8 + 2Pt(NH_3)_2 − 4NH_3]^{2−}$	1358.6462	1358.647279	–0.79	C77H95N22O49P7Pt2
$[a8 + 2Pt(NH_3)_2 − 3NH_3]^{2−}$	1367.15929	1367.160546	–0.92	C77H98N23O49P7Pt2
$[a8 + 2Pt(NH_3)_2 − 2NH_3]^{2−}$	1375.67363	1375.673813	–0.13	C77H101N24O49P7Pt2
$[a8 + 2Pt(NH_3)_2 − 1NH_3]^{2−}$	1384.18703	1384.18708	–0.04	C77H104N25O49P7Pt2
$[a9 − C9 + 2Pt(NH_3)_2 − 3NH_3]^{2−}$	1456.15984	1456.162144	–1.58	C82H105N23O54P8Pt2
$[a9 − C9 + 2Pt(NH_3)_2 − 2NH_3]^{2−}$	1464.67562	1464.675411	0.14	C82H108N24O54P8Pt2
$[a9 − C9 + 2Pt(NH_3)_2 − 1NH_3]^{2−}$	1473.18718	1473.188678	–1.02	C82H111N25O54P8Pt2
$[a9 − C9 + 2Pt(NH_3)_2]^{2−}$	1481.70272	1481.701945	0.52	C82H114N26O54P8Pt2
$[a9 + 2Pt(NH_3)_2 − 3NH_3]^{2−}$	1511.68401	1511.683781	0.15	C86H110N26O55P8Pt2
$[a9 + 2Pt(NH_3)_2 − 2NH_3]^{2−}$	1520.1961	1520.197049	–0.62	C86H113N27O55P8Pt2
$[a9 + 2Pt(NH_3)_2 − 1NH_3]^{2−}$	1528.71202	1528.710316	1.11	C86H116N28O55P8Pt2
$[w2]^{−}$	595.09621	595.096032	0.30	C18H26N6O13P2
$[w3]^{−}$	899.14205	899.142069	–0.02	C28H39N8O20P3
$[w3]^{2−}$	449.0674	449.0673965	0.01	C28H39N8O20P3
$[w4]^{−}$	1188.18859	1188.188441	0.13	C37H51N11O26P4
$[w4]^{2−}$	593.59076	593.5905825	0.30	C37H51N11O26P4
$[w5]^{2−}$	745.61388	745.613601	0.37	C47H64N13O33P5
$[w6]^{2−}$	897.63696	897.6366195	0.38	C57H77N15O40P6
$[w6]^{3-}$	598.0888	598.0886543	0.24	C57H77N15O40P6
$[w7]^{2-}$	1042.15936	1042.159806	–0.43	C66H89N18O46P7

$[w7]^{3-}$	694.43773	694.437445	0.41	C66H89N18O46P7
$[a3 - T3]^{-}$	675.12248	675.122247	0.34	C23H30N6O14P2
$[a5 - C5]^{-}$	1283.21407	1283.214321	−0.20	C43H56N10O28P4
$[a5 - C5]^{2-}$	641.10364	641.1035225	0.18	C43H56N10O28P4
$[a7 - T7]^{2-}$	937.6497	937.649727	−0.03	C62H81N15O41P6
$[a8 - G8]^{3-}$	726.11289	726.1127387	0.21	C72H94N17O48P7
$[a8 - G8]^{2-}$	1089.67257	1089.672746	−0.16	C72H94N17O48P7
$[a9 -C9]^{3-}$	835.79738	835.7969117	0.56	C82H106N22O54P8
$[a9 - C9]^{2-}$	1254.19822	1254.199006	−0.63	C82H106N22O54P8
$[C2: T7]^{3-}$	656.41958	656.4193913	0.29	C63H83N14O45P7
$[C2: T7]^{2-}$	985.13306	985.132725	0.34	C63H83N14O45P7
$[T3: T4 \text{ or } T6: T7 \text{ or } T10: T11]^{1-}$	785.08781	785.087908	−0.12	C25H33N4O19P3
$[C2: T6]^{2-}$	833.10995	833.1097065	0.29	C53H70N12O38P6
$[T3: T7]^{2-}$	840.60968	840.6095395	0.17	C54H71N11O39P6
$[a6]^{2-}$	848.64849	848.648172	0.37	C57H74N15O36P5
$[y6]^{2-}$	857.65376	857.6534545	0.36	C57H76N15O37P5
$[y7]^{2-}$	1002.17666	1002.176641	0.02	C66H88N18O43P6
$[a4]^{-}$	1105.21124	1105.211211	0.03	C38H49N10O23P3
$[C2: T4 \text{ or } C9: T11 \text{ or } T11: T13]^{-}$	1074.13396	1074.13428	−0.30	C34H45N7O25P4
$[C2: T4 \text{ or } C9: T11 \text{ or } T11: T13]^{2-}$	536.56367	536.563502	0.31	C34H45N7O25P4
$[T3: T6 \text{ or } T4: T7 \text{ or } T10: T13]^{2-}$	688.5868	688.5865205	0.41	C44H58N9O32P5
$[T3: T6 \text{ or } T4: T7 \text{ or } T10: T13]^{-}$	1378.17846	1378.180317	−1.35	C44H58N9O32P5
$[y3]^{-}$	819.17601	819.175739	0.33	C28H38N8O17P2

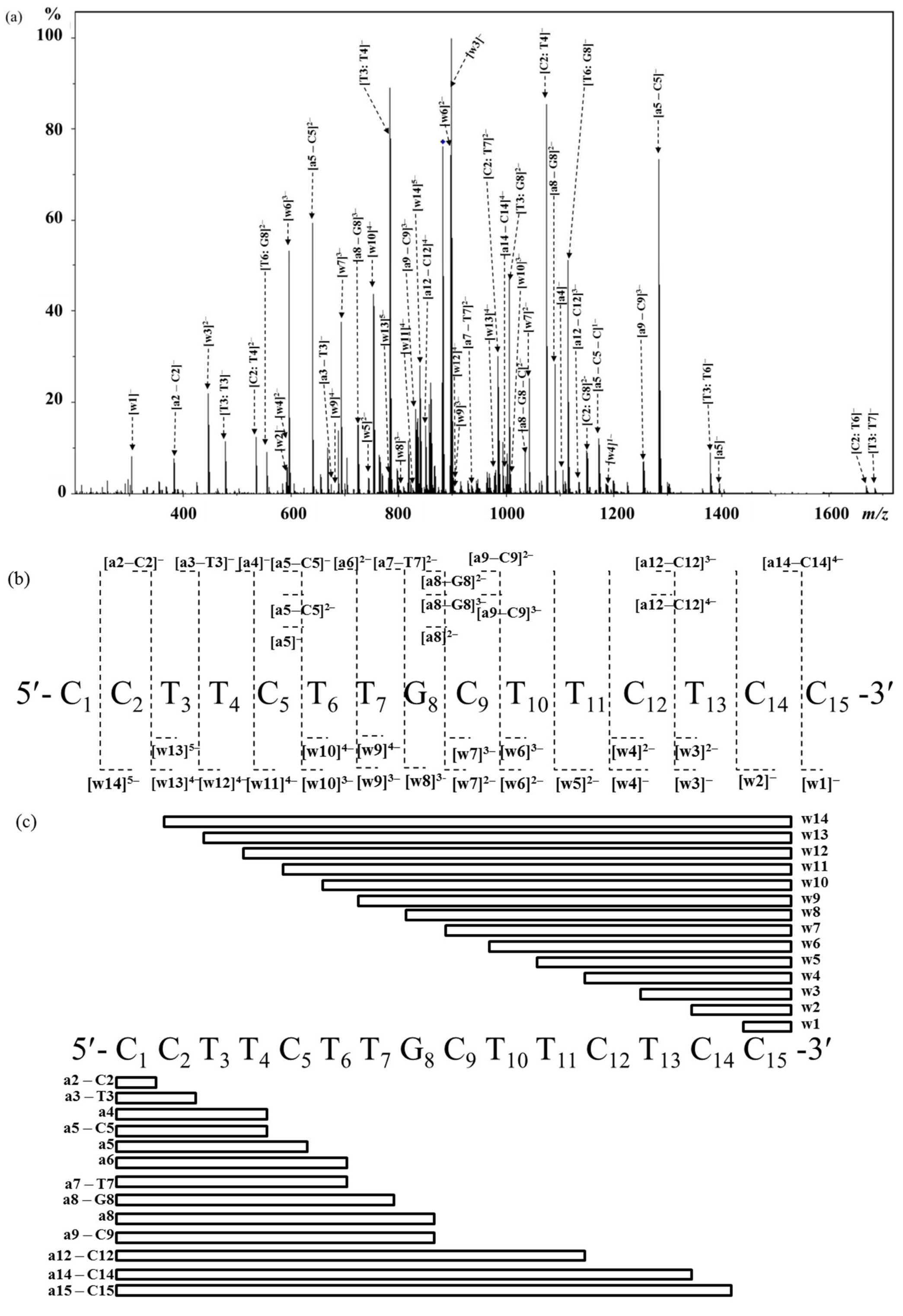

Figure S1. FT-ICR CID MS/MS spectrum of $[ODN15 - 5H]^{5-}$ (a), along with corresponding fragmentation maps (b) and fragments sequence of ODN15 (c). Main fragments were assigned and labeled in the spectrum shown in (a).
